# Perspectives of pharmaceutical stakeholders on determinants of medicines accessibility at the primary care level

**DOI:** 10.1186/s42506-020-00062-x

**Published:** 2021-01-13

**Authors:** Hoda Ibrahim Rizk, Monira Mahmoud Elkholy, Abeer Abdou Barakat, Raghda Mostafa Mostafa Elsayed, Shaimaa A. M. Abd El Fatah

**Affiliations:** Public Health and Community Medicine, Faculty of Medicine, Cairo University, 12 Manial Street, Cairo, Egypt

**Keywords:** Medicines, Access determinants, Pharmaceutical system, Stakeholders’ views

## Abstract

**Background:**

Equitable access to essential medicines of maintained efficacy, safety, quality, and cost-effectiveness must be ensured by a well-functioning health system. This study aims to identify the determinants of patients’ access to medicines at the primary health care (PHC) level from the perspectives of various (internal and external) stakeholders of the pharmaceutical system.

**Methods:**

The study employed both quantitative and qualitative components. Quantitative component applied a descriptive a cross-sectional design and qualitative component applied an in-depth interview design. It was a health system research conducted at two (PHC) facilities (one urban and the other rural) in Egypt. It inquired upon political, economic, and managerial aspects of the pharmaceutical system utilizing the “Health System Assessment Approach: a How-To Manual” and the “WHO operational package for assessing, monitoring and evaluating country pharmaceutical situations.”

**Results:**

Analysis of the quantitative data extracted from the cross-sectional component with external stakeholders (patients) revealed that about one-third of patients in both facilities were unable to pay for the medicine. Patients in both settings took less than an hour to reach the PHC facility. The Percent of patients who believe that the private pharmacies’ medicine is better than the PHC one was significantly higher in rural than urban group (24% and 10% respectively) and the percent of medicines dispensed was 50% and 66.7% in rural and urban groups respectively. Analysis of the qualitative data extracted from in-depth interviews with internal stakeholders (key informants from regulatory agencies, pharmaceutical industry, academia, pharmacists, and physicians) were summarized utilizing Strengths-Weaknesses-Opportunities-Challenges (SWOC) analysis approach. Various viewpoints toward the determinants of patients’ access to medicines were disclosed.

**Conclusions:**

The Percent of medicines dispensed was insufficient in both rural and urban facilities. There is a need to invest in building trust in generic medicine quality in the government health facilities focusing on improving medicine availability and ensuring enough amounts of high-quality drugs. Although there are drug committees in the two studied PHC facilities for demonstrating the prescribing and dispensing policies, yet the system required to enforce these policies is still deficient.

## Introduction

In an adequately functioning health system, essential medicines are proposed to be within the reach of individuals and communities at all times, in proper amounts, in the suitable dosage, with asserted quality, and an affordable price [1]. Access to medicines is included in the sustainable development goals (SDGs) target 3.8 which recognizes that guaranteeing universal health coverage, comprises access to affordable, safe, and effective essential medicines [2].

According to the pharmaceutical management framework, access is a construct of several dimensions: geographic accessibility, product availability, financial accessibility, and cultural acceptability [3]. It is venerable to embrace in-country stakeholders in all phases of access evaluation from making up the work plan through managing the assessment, spreading, and validating the findings and recommendations [4].

Egypt’s total pharmaceutical spending accounts for 1.89% of the GDP and constitutes 34.20% of the total health disbursement. The percentage of public expenditure on health declined from 5.04% in 2011/2012 to 4.3% in 2018/2019; this makes Egypt’s health expenditures one of the lowest in the Arab region [5].

Egypt, like many developing health systems, cannot simply measure their constraints and lassitude, which makes policy-makers unenlightened regarding scientifically correct information of what they can and should strengthen. In such weak systems, corrective interventions—even the plain ones—often do not accomplish their objectives [6].

In our study, issues preventing “access to medicines for all” need to be explored and addressed if we are to ensure that there is universal access to this human right.

This study aims to identify the determinants of patients’ access to medicines at the primary health care (PHC) level from the perspectives of various (internal and external) stakeholders of the pharmaceutical system.

## Methods

### Study setting

Two PHC units in Giza Governorate were selected to be the study setting for the following reasons: Giza governorate is within the catchment area of Cairo University, and the Faculty of Medicine mission has a social and health responsibility toward the catchment area community. Also, according to 2017 census, Giza Governorate population size was 8,759,000 and it is composed of 19 districts that include urban and rural areas [5]. Kafr-Tohormos unit (was selected to represent the rural area) and Abou-Ragwan unit (to represent the urban area) at Giza Governorate.

### Study design

This study included both qualitative and quantitative components. The quantitative component applied a descriptive cross-sectional design and the qualitative component applied an in-depth interview design. We used the approach of stakeholders’ views parsing through Strengths, Weaknesses, Opportunities and Challenges (SWOC) analysis.

### Study population

**Fig. 1:**
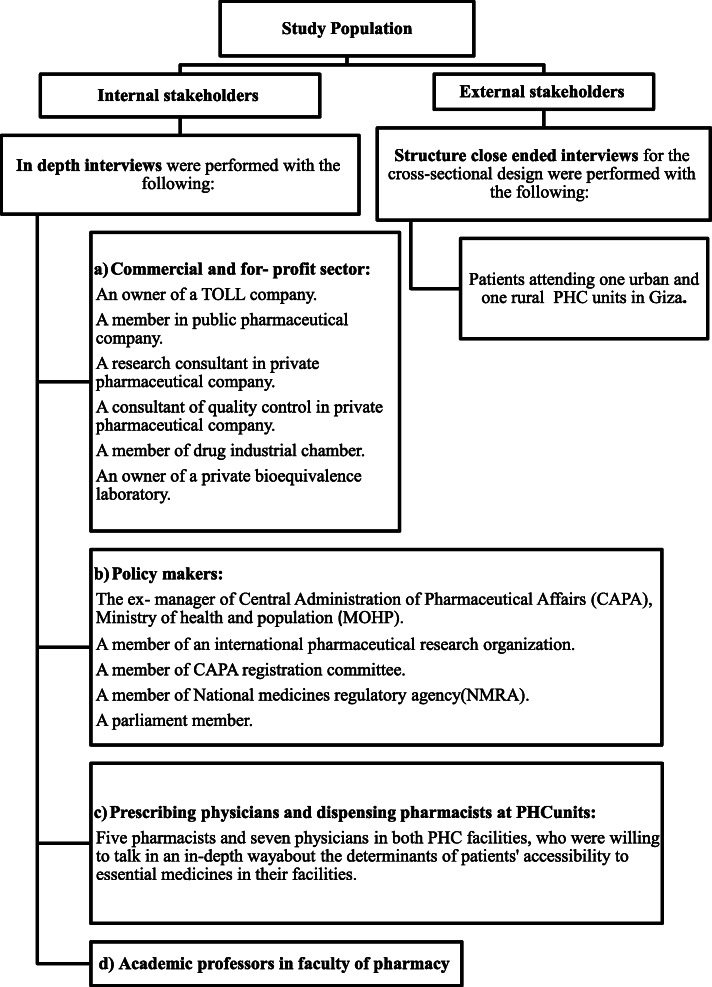
Internal and external stakeholders of the pharmaceutical system

Both internal and external stakeholders of the pharmaceutical system at Giza Governorate were included (Fig. 1).

### Sampling size and technique

#### For the quantitative component

According to The Ministry of Health and Population (MOHP) records, in the year preceeding the study, the number of attendants to either the studied urban or the rural PHC unit was estimated to be 10% of the total population, which is nearly 100,000 citizens per unit [5]. The sample size calculation was done using (Epi Info™) for cross-sectional studies. Based on a statistical power of 95% confidence level, 5% margin of error, and 10% response rate, the minimum recommended size from the rural unit was 138 patients and the same number from the urban unit [7]. Any patient who received services from the PHC units and approved to participate in this survey was included in the study regardless of age or sex. Data collection was done over a period of 6 months starting from January 2019 to June 2019.

#### For the qualitative component

Due to the difficult access to stakeholders in the field of pharmaceuticals, the snowballing technique was adopted [8]. The researchers started with an interviewee and at the end of the interview, he/she was asked to allow contact with a new interviewee. The size of the sample in case of in-depth interviews was “1-2 of each category.”

#### Data collection

Data was collected by questionnaire (cross-sectional part) and by in-depth interview guide utilizing the Health System Assessment Approach: a How-To Manual [9], and the WHO operational package for assessing, monitoring and evaluating country pharmaceutical situations [10].

#### Qualitative component

An in-depth interview guide was prepared and utilized with variant stakeholders [9, 10]. Interviews were done with twenty-five interviewees at their workplace. Open-ended questions were used to explore their different perspectives and experiences in the field of pharmaceuticals (Table 1).

**Table 1:** In-depth interview guide with variant stakeholders

Stakeholders	Open-ended questions
**a) The commercial and for-profit sector** **b) Policymakers** **c) Academic professor in faculty of pharmacy** **d) WHO consultant**	• Existence of a national medicines regulatory agency (NMRA) responsible for the promulgation and enforcement of regulations. • Existence of a system for pharmaceutical registration. • Existence of a pharmacovigilance system. • Mechanisms exist for licensing, inspection, and quality control. • Views about different pricing policies. • Production and trade.
**Pharmacists**	• Knowledge about the essential drug list (EDL). • Existence of functioning mechanisms to improve dispensing practices. • Existence of drug committee and what is its function. • Opinion about the quality of generic versus brand. • Percent of prescribed medicines from EDL. • Percent of medicines prescribed by generic name. • The percent availability and the average period of stock-outs of unexpired essential tracer medicines.
**Physicians**	• Prescribing behavior of physicians • Perception of affordability • Attitudes of physicians toward generic drugs prescribing • Relation to medical representatives • Perception of EDL policies • Perception and commitment to guidelines’ policies • Perception and suggestions about pharmaceutical management

#### Quantitative component

PHC facility attendants were invited for interviews (exit interview) using a structured questionnaire with closed-ended questions prepared by the investigators according to the Health System Assessment Approach and the WHO operational package [9, 10]. It included the following:

- Demographic and socioeconomic background.
- Cause of attendance, prescribed medication, availability, and suitability of its price.
- Geographical access to essential drug list (EDL): residing within 1 h walk.
- Financial access to EDL: ability to pay, willingness to pay, perception about drug price, public co-payments versus private fees. This part is guided by Egypt Household Health Expenditure and Utilization Survey [11].
- Cultural barriers: public and generic drugs.
- Drug availability: frequency of visits with unavailable drugs in public stores.

#### Data management and statistical analysis

##### Quantitative data analysis

- Data were analyzed using Statistical Package for Social Science (SPSS) program, IBM SPSS Statistics for Windows, Version 25.0. (Armonk, NY: IBM Corp).
- Tests of normality of data (like Kolmogorov-Smirnov test) revealed that data was not normally distributed. That is why appropriate statistical tests of significance were used to test the null hypothesis in the comparison between groups. Non-parametric tests like Mann-Whitney test were used in univariable comparisons to quantify the associations of continuous variables while the chi-square test was used for categorical variables. The difference between groups was considered significant at *P* value < 0.05

##### Qualitative data analysis

The thematic content analysis method was applied after ensuring that no unprecedented data came out, and that data reached to saturation[12]. Analysis of data extracted from in-depth interviews with internal stakeholders was summarized utilizing (SWOC) analysis approach.

## Results

The cross-sectional component included 276 patients from urban and rural residential areas in Giza Governorate. Although there was no significant difference between rural and urban residents concerning education and occupation yet there was a significant difference concerning the median overall monthly income (Table 2).

**Table 2:** Factors associated with patients’ financial accessibility to the essential drug list among rural and urban groups attending PHC units, Giza Governorate, Egypt, 2019

Financial accessibility	Residence		***P*** value*
	Rural unit (***N*** = 138) (%)	Urban unit (***N*** = 138) (%)	
**Willingness to pay for the medicine**			
Yes	100 (72.4)	112 (81.1)	0.133
No	38 (27.6)	26 (18.9)	
**Ability to pay for the medicine**			
Yes	98 (71.1)	87 (63.1)	0.303
No	40 (28.9)	51 (36.9)	
**Coping mechanisms**			
**Borrowing to buy the medicine**			
Yes	21 (15.3)	21 (15.3)	1
No	117 (84.7)	117 (84.7)	
**Selling assets to buy the medicine**			
Yes	7 (5.1)	4 (2.9)	0.721
No	131 (94.9)	134 (97.1)	
**Out of pocket expenses** (Egyptian pounds L.E.)			
	**Median (IQR)**	**Median (IQR)**	***P*** value**
**Expenses on medicine in the current visit**	15.75 (5‑73)	23.5 (2‑103)	˂ 0.001
**Expenses on medicine/month**	200 (20‑500)	100 (8‑550)	0.056
**The estimated income per family member**	222.2 (75‑1000)	333.3 (83.3‑2250)	˂ 0.001

*P value was calculated using chi-square test

**P value was calculated using the Mann-Whitney test

*P* value is statistically significant < 0.05

The median expenses paid by patients to have the medicine in their visit to the PHC facility were significantly higher in the urban group than the rural group (*P* value ˂ 0.001). However, no significant differences were detected between rural and urban residents concerning willingness to pay or the ability to pay for the medicine or expenses paid on medicine per month. Both rural and urban groups cope more or less in the same manner to buy medicine (Table 2).

Participants’ belief that private pharmacies’ drugs are better than PHC drugs was significantly higher in the rural group compared to the urban group (*P* = 0.009). The majority of participants in rural and urban areas take less than an hour to reach the PHC facility (99%, 100% respectively) with no significant difference between them. The number of visits in the previous 3 months was significantly higher in the rural group in comparison to the urban group (Table 3).

**Table 3:** Product availability, cultural acceptability, and geographical accessibility to PHC units providing essential drug list to rural and urban groups during the study period (2019)

Product availability	Residence		***P*** value**
	Rural unit	Urban unit	
	Median (min.‑max.)	Median (min.‑max.)	
**No. of drugs prescribed**	2 (1‑7)	3 (1‑8)	˂ 0.001
**No. of drugs dispensed**	1 (0‑3)	2 (0‑6)	˂ 0.001
**Percent of drugs dispensed**	50 (0‑100)	66.7 (0‑100)	0.984
**No. of visits in the previous 3 months**	4 (1‑15)	3 (1‑20)	0.012
**No. of visits during which patients found all the prescribed drugs**	1 (0‑9)	1 (0‑15)	0.552
**Percent of visits where all drugs were found**	27.5 (0‑100)	33.3 (0‑100)	0.645
**Geographical accessibility**			
	**(*N*** **= 138) (%)**	**(*N*** **= 138) (%)**	***P*** value*
**Less than an hour**	137 (99.3)	138 (100)	1
**More than an hour**	1 (0.7)	0 (0)	
**Cultural acceptability**			
**A belief that herbal treatment is better than drugs**			
Yes	42 (30.4)	31 (22.5)	0.197
No	96 (69.6)	107 (77.5)	
**A belief that private pharmacies drugs are better than PHC drugs**			
Yes	33 (23.9)	14 (10.2)	0.009
No	105 (76.1)	124 (89.8)	

*P value was calculated using chi-square test

**P value was calculated using the Mann-Whitney test *P* value is statistically significant < 0.05

The median number of drugs prescribed and dispensed for patients in the urban PHC facility was significantly higher than the rural one (Table 3).

**Fig. 2:**
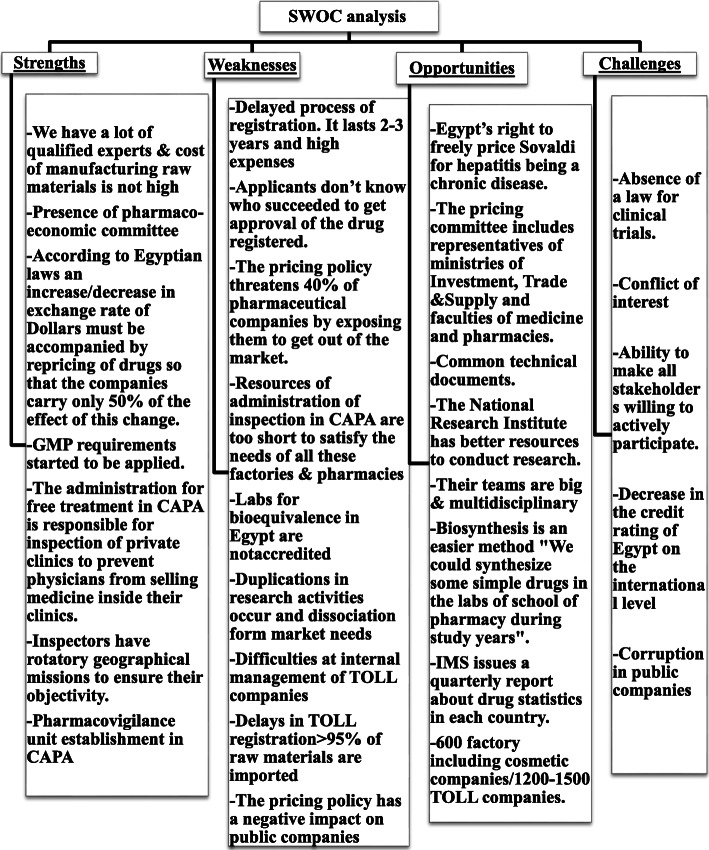
Summarization of Strengths, Weaknesses, Opportunities and Challenges (SWOC) analysis done utilizing the available qualitative data

The purposive recruitment for in-depth interviews helped in disclosing various viewpoints toward the determinants of patients’ access to medicines (Fig. 2).

The researchers came into the following themes while analyzing the qualitative data extracted from the in-depth interviews.

### Perspectives of key informants from regulatory agencies, pharmaceutical industry were arranged under the following themes

The nature of legal provisions for registration, causes of delay, pricing policies, public price of a drug, drugs shortage, pharmaceutical regulation (counterfeit drugs), pharmaco-vigilance, effectiveness of MOHP drugs in comparison to private market drugs, research and development role, drug promotion, medicines production capability in the country, TOLL Companies, and industry (Additional file 1).

### Perspectives of physicians and pharmacists toward determinants of patients’ access to EDL were arranged under the following themes

Prescribing behavior, affordability, generic drugs prescribing, medical representatives, commitment to EDL guidelines policies, managerial control on prescribing behavior, positive and negative aspects of pharmaceutical management in PHC, percent availability of essential tracer medicines stock-outs (Additional file 1).

Eventually, recommendations of both key informants and physicians-pharmacists for improving access to medicines were cited and greatly regarded (Additional file 2).

## Discussion

While assessing determinants of patients’ financial accessibility, medicines availability, cultural acceptability, and geographical accessibility to facilities providing essential drugs among rural and urban populations, we were attentive to achieve stakeholder inclusiveness using a good and balanced choice.

The current study revealed that one-third of patients who did not get the prescribed drugs were unable to pay for the medicine. Donadel and his colleagues performed a study at PHC level in Tajikistan, 2016, where one-third of the interviewees who did not take in the prescribed drugs related it to their inability to pay. Their results are in the same direction as ours [13].

As regards medicine availability, the results of the present study goes in concordance with a study done in 36 developing and middle-income countries about medicine availability and concluded that the average public sector availability of medicines ranged from 29.4 to 54.4% [14]. Also, a study performed by Nascimento et al., 2017 showed that around 60% of patients said they pick up their needed medicines from PHC units [15].

However, a study conducted in PHC centers in Pakistan found that 90.9% of the prescribed drugs were dispensed [16]. Usually, the low percentage of actually dispensed drugs could be mainly attributed to inadequate drug stock. But also, essential drugs constrained availability is sometimes connected with budgetary restraints, fractional drug supply system, or indigent inventory management of the responsible staff [14, 15].

Concerning cultural acceptability, our observations effectuate the findings of many studies that investigated patients’ knowledge of generic medicines. They revealed that patients are inclined to branded medications as they think that medicine in private pharmacies is better than medicine in PHC facilities [17–19]. Patients felt that generic medicines were subsidiary and that herbal treatment is better. This could be attributed to their explanation that herbal treatments carry less chemicals and are less anticipated to bring adverse reactions [18].

The number of medicines per prescription (NMPP) is a prescribing indicator that is pertinent to the rational use of medicine (RUM). A high NMPP can stamp irrational use practices [19]. Regarding the percent of medicine prescribed from EDL, our results are analogous to a similar study, which found that the drug commanded from the EDL in urban PHC was above 90 % [16].

Our findings are consistent with a study by Gopalakrishnan et al., 2013, where it was found that nearly 71% of urban doctors were prescribing the drugs by generic names, but only 52% of the rural doctors did that [20]. Impairing access of poor citizens to health care occurs when patients buy expensive drugs instead of generics. Unfortunately, this happens when doctors prescribe brand names instead of International non-proprietary names (INN) [13].

Measuring physicians’ performances in terms of RUM does not only include contents of the prescriptions but also entails the cost of the prescribed medicines. In our study, most physicians mentioned that they put the cost of the drug into consideration. It was found that median expenses paid by patients to have the medicine in their visit to the PHC facility were significantly higher in the urban group than the rural group; this difference could be explained by the fact that patients in urban units often pay part of the cost of medicine.

This comes with the fact that nearly three quarters of Egypt’s health spending (72%) came directly from household out-of-pocket (OOP) payments [11]. Although the cost per prescription (CPP) prices of countries can yield foresight concerning pharmacoeconomic evaluations, yet it is impracticable to objectively compare the CPP prices of different countries because of the variations in purchasing power [19].

In similar studies, physicians reported that patient variables like clinical condition, compliance, financial situation, and ability to purchase had a powerful influence on their prescribing decisions. Meanwhile, the activities of pharmaceutical companies have little or no influence [21, 22]. In our study, physicians mentioned they did not respond to patient pressure to prescribe non-indicated drugs. On the contrary, another study denoted that patients and their families applied different kinds of direct and indirect pressures to affect physicians’ decisions as regards their prescription [22]. Physicians in the current study might have been reluctant to express their true opinions to the interviewer.

Sometimes nonessential drugs are prescribed to patients just to fulfill patients’ wrong awareness of health care. The enlightenment of common people and health professionals about the dangers of careless curative care is a distinct demand for modern times. Even in different countries, with the same level of health care expenditure, only diminution of clinical health care minimizes costs for the public health system and makes it more effective [20].

Research suggests that working constraints like time, infrastructure, and service management limit the provision of sound, user-oriented dispensing services. Sometimes the pharmaceutical practice actions are complex and the bad service level can result in impactful dispensing errors for the patients’ health [23]. Though there is a drug committee in the two studied PHC facilities for demonstrating the prescribing and dispensing policies, the system required to enforce these policies is still deficient. Duong et al. has suggested that in order to alleviate medicine shortages and to enable fair and equitable access to essential medicines, all committee members must clearly organize their decision-making priorities with procurement practices. Measures like prioritization of supply (number and diversity of product choices), storage space, ordering, transportation, budget decisions, and resilience planning in cases of unpredictable circumstances, such as expanded consumer demand, all secure sustainable patient care [24]. In order to improve drug committee function, there is a need to expand the roles of pharmacists and wholesaler/distributors in the committee decisions to improve the technique of medicines selection and procurement [25].

The current study revealed that Egypt’s reference price is the public one. This is consistent with a study performed by Kaló et al. on how external reference pricing (ERP) is extensively used in the Middle East. ERP rules are most strict in Egypt and Saudi Arabia; they dictate the least pharmaceutical price out of a basket containing more than 25 countries each. On the contrary, Kuwait and Qatar reference only the country of origin for new pharmaceuticals and they do not routinely use EPR for revision of prices after initial pricing decisions [26]. Egyptian MOHP, till 2012, adopted a cost-plus procedure to put the retail prices based on cost reports from producers when products were commenced to be sold in the Egyptian market. Once set, these prices were seldom re-evaluated to adjust for any alterations in cost from inflation or exchange rate oscillation. Hence, retail prices often persisted unvarying over several years and have been heeded very low by manufacturers [27].

In many Asian, African, and Latin American countries, certain strategies were used to contain pharmaceutical prices. The most commonly used pricing policy was mark-up regulation, followed by ERP, cost-plus, and finally generic promotion. The least used policy was the tax exemption [28]. ERP policy uses benchmarking against a basket of countries. Its evident limitation included that optimal ERP should be benchmarked against countries with an analogous economic position [26]. A study in Lebanon and Egypt where prices are benchmarked against developed and high-income countries (HICs) showed that this policy resulted in higher medicine prices for local clients, the requirement of proficient teams for policy design, implementation, and lack of transparency in results appraisal and decision-making [29]. In many low- and middle-income countries (LMICs), the reference price did not often become the actual national price due to the lack of precise analytical studies or monitoring reports. Moreover, companies responded by minimizing price transparency, resulting in the unevenness of the ERP mechanism [30].

Concerning pharmacovigilance (PV), our study demonstrated that there is a unit established in Egypt’s central administration of pharmaceutical affairs (CAPA). Of twenty-six sub-Saharan African countries, only eight countries collected reports on adverse events. Among them, only 3 programs were qualified to grant a sizeable number of PV reports [19]. Determining the performance of PV systems in a country requires membership of this country to the WHO—Program for International Drug Monitoring (PIDM). This membership requires a dedicated national PV center, an automatic adverse drug reaction (ADR) reporting system, and technical efficiency in managing individual case safety reports (ICSRs) [31].

A problem facing the National Medicine Regulatory Agency (NMRA) in most developing countries is the lack of human resources needed to conduct the inspection due to low salaries, insufficiency of pharmaceutical and training institutions [32]. Globally, the WHO rates that at least 30% of NMRA worldwide have confined the ability to accomplish the core regulatory functions [19]. The evaluation of bioequivalence data often presents a major challenge (technical and financial) for regulatory authorities in LMICs including Egypt to the extent that only some local manufacturers, not all of them can carry out bioequivalence studies [33, 34].

Concerning registration, our study findings are consistent with a South African study where registration timelines ranged between 1 and 3 years. Advancement of access to new medicines, through emboldening more companies to register medications in Africa could only be done through a proficient, foretold registration timeline [35]. Another study on marketing authorization (MA) demonstrated that market availability of the drugs is influenced by the time taken by an applicant to be granted an MA certificate [19]. Other causes of turning down were the potential conflicts of interest recorded in at least three African countries and the extra fees rushed upon for initial MA and provisions for its renewal certificate. Studies among both local and multinational pharmaceutical companies in Africa revealed that registration prices were among reasons for companies in making decisions not to provide medicines to specific countries [36].

Our findings are consistent with other study, which showed that the international community does not back up local pharmaceutical production in Africa. Certain African governments believe that investing in the local industry is a bad use of funds and that international procurement of expensive drugs is a more skillful option [32]. In the short run, importing instead of manufacturing appears like a faultless choice but it is a wrong notion in the long run as dependence on imports comes out with a contingent increase in prices [37].

In the Arab region, the local drug production builds up from 0% up to more than 90% of national drug outlets. The Egyptian marketplace in certain studies has been rated to be nearly US$ 1.7 billion. Formulations represent the most locally produced drugs. In some Arab countries, multinational companies give a production license to about 40% of drugs. The imported raw materials needed for local production reaches more than 90% [38].

The research and development activities in the drug industry are primarily quality control activities [37]. Initiation of the manufacturing of biotechnology products, especially vaccines is the focus of recent efforts in Egypt and other Arabic countries. Lack of funding, lack of experienced human resources in certain specialized areas, and lack of suitable environment represent the major challenges facing research and development [39]. Twelve percent of samples collected from Egypt, in a multi-country medicine quality survey, failed at minimum one medicine quality test and can be heeded substandard [40].

### Limitations

Performing the study in a single governorate only was done owing to difficult administrative permissions. A second limitation was the inability to check the expiry date on tracer medicines as the PHC staff considered that as a sensitive issue. Difficulty in interviewing these various stakeholders and in taking appointments may have affected the quality of information given by the informants.

## Conclusion

The study concluded that about one third of patients in both urban and rural facilities were unable to pay for the medicine. Though there is a drug committee in the two studied PHC facilities for demonstrating the prescribing and dispensing policies, yet the system required to enforce these policies is still deficient. Building trust in generic medicine quality, as well as PHC in government health units must be the focus of future programs for improving medicine availability.

The evaluation of bioequivalence data often presents a major challenge (technical and financial) for regulatory authorities. Favoring high-quality locally manufactured medicines can be simply forwarded by enhancing incentives like publishing internal and external audits of drug regulatory agencies, close monitoring of medicines dealers, ameliorating the capability of the national regulatory authority to effectuate necessities, transparency, and accountability of the inspection process.

## Supplementary Information

**Additional file 1.** I- Views of stakeholders toward pharmaceutical registration. II- Perspectives of physicians toward determinants of patients’ access to essential drug list. III- Perspectives of pharmacists toward determinants of patients’ access to essential drug list.

**Additional file 2.** Suggestions of key informants for improving access to medicines. Suggestions of physicians and pharmacists for improving drug affordability.

## Data Availability

The quantitative datasets used and/or analyzed during the current study are available from the corresponding author on reasonable request. All qualitative data generated or analyzed during this study are included in this published article [and its supplementary information files].

## Abbreviations

ADR: Adverse drug reaction
CAPA: Central administration of pharmaceutical affairs
CPP: Cost per prescription
GDP: Gross domestic product
EDL: Essential drug list
EGP: Egyptian pound
ERP: External reference pricing
HIC: High-income countries
INN: International non-proprietary names
ICSRs: Individual case safety reports
LMICs: Low- and middle-income countries
MA: Marketing authorization
MOHP: Ministry of health and population
NMPP: Number of medicines per prescription
NMRAs: National medicines regulatory agencies
OOP: Out of pocket
PHC: Primary health care
PIDM: Program for international drug monitoring
PV: Pharmacovigilance
RUM: Rational use of medicine
SDGs: Sustainable development goals
SPSS: Statistical Package for Social Science
SWOC: Strengths, Weaknesses, Opportunities, and Challenges
WHO: World Health Organization

## References

[1] World Health Organization (WHO): Essential medicines. WHO. 2019 https://www.who.int/medicines/services/essmedicines_def/en/.

[2] United Nations (UN). Sustainable development goals. 2016. https://www.un.org/sustainabledevelopment/sustainable-development-goals/.

[3] Bigdeli M, Jacobs B, Tomson G, Laing R, Ghaffar A, Dujardin B, et al. Access to medicines from a health system perspective. Health Policy Plan. 2013;28:692–704. 10.1093/heapol/czs108 PMC3794462 23174879

[4] Kraiselburd S, Yadav P. Supply chains and global health: an imperative for bringing operations management scholarship into action. Prod Oper Manage. 2013;22:377–381.

[5] Central Agency For Public Mobilization And Statistics (CAPMAS). Egypt in figures. Health. 2017. https://www.capmas.gov.eg/Pages/StaticPages.aspx?page_id=5035.

[6] Yazdi-Feyzabadi V, Bahrampour M, Rashidian A, Haghdoost AA, Akbari Javar M, Mehrolhassani MH. Prevalence and intensity of catastrophic health care expenditures in Iran from 2008 to 2015: a study on Iranian household income and expenditure survey. Int J Equity Health. 2018;17:44. 10.1186/s12939-018-0743-y PMC5899413 29653568

[7] Epi Info™ version 7.2.2.16., a database and statistics program for public health professionals. CDC, Atlanta, GA, USA, 2018.

[8] Creswell JW, David CJ. Research design: qualitative, quantitative, and mixed methods approaches. Fifth edition: SAGE publications; 2017.

[9] United States Agency International Development (USAID). The health system assessment approach: a how-to Manual. Version 2.0. 2012. https://www.hfgproject.org › HSAA_Manual_Version_2_Sept_20121.

[10] World Health Organization (WHO). WHO operational package for assessing, monitoring and evaluating country pharmaceutical situations, Guide for coordinators and data collectors 2007. https://www.who.int/iris/handle/10665/69927 ;2007.

[11] Rafeh, Nadwa, Julie Williams, Nagwan Hassan. Egypt Household Health Expenditure and Utilization Survey 2010. Bethesda: Health Systems 20/20 project, Abt Associates Inc. 2010. https://www.hfgproject.org › egypt-household-health-expenditure-utilizati.

[12] Luciani M, Orr E, Campbell K, NGuyen L, Ausili D, Jack SM. How to design a qualitative health research study. Part 2: data generation and analysis considerations. Professioni Infermieristiche. 2019 72(3):221-231. . 31883573

[13] Donadel M, Karimova G, Nabiev R, Wyss K. Drug prescribing patterns at primary health care level and related out-of-pocket expenditures in Tajikistan. BMC Health Serv Res. 2016;16:556. 10.1186/s12913-016-1799-2 PMC5053171 27716266

[14] Cameron A, Ewen M, Ross-Degnan D, Ball D, Laing R. Medicine prices, availability, and affordability in 36 developing and middle-income countries: a secondary analysis. Lancet. 2009;373(9659):240–249. 10.1016/S0140-6736(08)61762-6 19042012

[15] Nascimento RCRM, Alvares J, Guerra Junior AA, Gomes IC, Costa EA, Leite SN, et al. Availability of essential medicines in primary health care of the Brazilian Unified Health System. Rev Saude Publica. 2017;51(Suppl 2):10s. 10.11606/S1518-8787.2017051007062 PMC5676352 29160448

[16] Atif M, Sarwar MR, Azeem M, Umer D, Rauf A, Rasool A, et al. Assessment of WHO/INRUD core drug use indicators in two tertiary care hospitals of Bahawalpur, Punjab, Pakistan. J Pharm Policy Pract. 2016;9:27. 10.1186/s40545-016-0076-4 PMC5034517 27688887

[17] Aivalli PK, Elias MA, Pati MK, Bhanuprakash S, Munegowda C, Shroff ZC, et al. Perceptions of the quality of generic medicines: implications for trust in public services within the local health system in Tumkur, India. BMJ Glob Health. 2018;2:e000644. 10.1136/bmjgh-2017-000644 PMC5844374 29531844

[18] Barry AR. Patients’ perceptions and use of natural health products. Can Pharm J (Ott). 2018;151:254–262. 10.1177/1715163518779409 PMC6141934 30237840

[19] World Health Organization (WHO). Assessment of medicines regulatory systems in sub-Saharan African countries. Geneva: WHO Press; 2010. http://apps.who.int/medicinedocs/documents/s17577en/s17577en.pdf.

[20] Gopalakrishnan S, Ganeshkumar P, Katta A. Assessment of prescribing practices among urban and rural general practitioners in Tamil Nadu. Indian J Pharmacol. 2013;45:252–257. 10.4103/0253-7613.111931 PMC3696296 23833368

[21] Sharifnia SHA, Mohammadzadeh M, Arzani G, Salamzadeh J, Abolfazli SA, Zali A, et al. Main factors affecting physicians’ prescribing decisions: the Iranian experience. Iran J Pharm Res. 2018;17:1105–1115. PMC6094427 30127833

[22] Strumiło J, Chlabicz S, Pytel-Krolczuk B, Marcinowicz L, Rogowska-Szadkowska D, Milewska AJ. Combined assessment of clinical and patient factors on doctors’ decisions to prescribe antibiotics. BMC Fam Pract. 2016;17:63. 10.1186/s12875-016-0463-6 PMC4891944 27255505

[23] Leite SN, Bernardo NLM, Álvares J, Gurra Junior AF, Costa EA, Acurcio FA, et al. Medicine dispensing service in primary health care of SUS. Rev Saude Publica. 2017;51(suppl 2):11s. 10.11606/S1518-8787.2017051007121 PMC5676395 29160457

[24] Duong MH, Moles RJ, Chaar B, Chen TF. Stakeholder perspectives on the challenges surrounding management and supply of essential medicines. Int J Clin Pharm. 2019;41(5):1210–1219. 10.1007/s11096-019-00889-1 31444686

[25] Duong MH, Moles RJ, Chaar B, Chen TF. Stakeholder roles in facilitating access to essential medicines. Res Social Adm Pharm. 2019;15(3):260–266. 10.1016/j.sapharm.2018.04.034 29752050

[26] Kalό Z, Alabbadi I, Al Ahdab OG, Alowayesh M, Elmahdawy M, Al-Saggabi AH, Kanavos P. Implications of external price referencing of pharmaceuticals in Middle East countries. Expert Rev Pharmacoecon Outcomes Res. 2015;15:993–998. 10.1586/14737167.2015.1048227 26088919

[27] International Society for Pharmacoeconomics and Outcomes Research (ISPOR). Egypt pharmaceutical. Global Health Technology Assessment Road Map. https://tools.ispor.org › htaroadmaps › EgyptPH.

[28] Zhou Z, Syu Y, Campbell B, Zhou Z, Gao J, Yu Q, Pan Y. The financial impact of the ‘zero-markup policy for essential drugs’ on patients in county hospitals in western rural China. PLoS One. 2015;10:e0121630. 10.1371/journal.pone.0121630 PMC4366182 25790443

[29] Hammad EA. The use of economic evidence to inform drug pricing decisions in Jordan. Value Health. 2016;19:233–238. 10.1016/j.jval.2015.11.007 27021758

[30] Espin J, Rovira J, Olry de Labry A. WHO/HAI project on medicine prices and availability review series on pharmaceutical pricing policies and interventions working paper 1: external reference pricing 2011. http://haiweb.org/wp-content/uploads/2015/07/Working-Paper-1-External-Reference-Pricing.pdf.

[31] Ampadu HH, Hoekman J, de Bruin ML, Pal SN, Olsson S, Sartori D, et al. Adverse drug reaction reporting in Africa and a comparison of individual case safety report characteristics between Africa and the rest of the world: analyses of spontaneous reports in Vigi Base. Drug Saf. 2016;39:335–345. 10.1007/s40264-015-0387-4 PMC4796322 26754924

[32] Nayyar GM, Attaran A, Clark JP, Culzoni MJ, Fernandez FM, Herrington JE, et al. Responding to the pandemic of falsified medicines. Am J Trop Med Hyg. 2015;92(6 Suppl):113–118. 10.4269/ajtmh.14-0393 PMC4455086 25897060

[33] Galgatte UC, Jamdade VR, Aute PP, Chaudhari PD. Study on requirements of bioequivalence for registration of pharmaceutical products in USA, Europe and Canada. Saudi Pharm J. 2014;22:391–402. 10.1016/j.jsps.2013.05.001 PMC4246407 25473327

[34] Midha KK, McKay G. Bioequivalence; its history, practice, and future. AAPS J. 2009;11:664–670. 10.1208/s12248-009-9142-z PMC2782076 19806461

[35] Narsai K, Williams A, Mantel-Teeuwisse AK. Impact of regulatory requirements on medicine registration in African countries-perceptions and experiences of pharma companies in South Africa. South Med Rev. 2012;5:31–37. PMC3471191 23093897

[36] Ahonkhai V, Martins SF, Portet A, Lumpkin M, Hartman D. Speeding access to vaccines and medicines in low- and middle income countries: a case for change and a framework for optimized product market authorization. PLoS One. 2016;11:e0166515. 10.1371/journal.pone.0166515 PMC5112794 27851831

[37] Ahen F, Salo-Ahen HMO. Governing pharmaceutical innovations in Africa: inclusive models for accelerating access to quality medicines. Cogent Medicine. 2018;5:1500196.

[38] Al-Shakka M, Abood E, Al-Dhubhany A, Aldobei S, Said K, Jha N, Shankar PR. Problems facing the Arab pharmaceutical industry: a case study from Yemen. Res Pharm Healt Sci. 2016;2:234–241.

[39] World Health Organization (WHO). EMRO: regional health observatory data repository. WHO: Regional Office for the Eastern Mediterranean; 2015. http://www.emro.who.int/entity/statistics/regional-health-observatory.html.

[40] Khazzaka M. Pharmaceutical marketing strategies’ influence on physicians’ prescribing pattern in Lebanon: ethics, gifts, and samples. BMC Health Serv Res. 2019;19(1):80. 10.1186/s12913-019-3887-6 PMC6354386 30700295

