# Supplementary material for: Perspectives of pharmaceutical stakeholders on determinants of medicines accessibility at the primary care level
Source: J Egypt Public Health Assoc. 2021 Jan 13;96:1. doi: 10.1186/s42506-020-00062-x (PMC7806678; doi:10.1186/s42506-020-00062-x)
Supplement: Supplementary file 1 — Additional file 1. I- Views of stakeholders toward pharmaceutical registration. II- Perspectives of physicians toward determinants of patients’ access to essential drug list. III- Perspectives of pharmacists toward determinants of patients’ access to essential drug list. [file 42506_2020_62_MOESM1_ESM.docx]

**Appendix1:**

**I- Views of stakeholders toward pharmaceutical registration**

**_Regarding the nature of legal provisions for registration**: *"Registration of pharmaceuticals in Egypt follows what is called a “box” system. The number of similar products for any active pharmaceutical ingredient (API )in the specific dosage form is 12products called "Box" and is composed of one brand product, and 11 generic products (10locally manufactured generic products and 1 imported generic product)* said a member of National medicines regulatory agency (NMRA).

**_As regards the average turnaround time,** an interviewee said that there is a delayed process of registration *"It lasts 2-3 years while it could be managed within 7 months"* said a member of the registration committee.

**_As concerning the causes of this delay**, many interviewees highlighted that: *“for a drug to be registered it should pass through many processes. Each process is managed by a committee of experts. Involved processes include: Technical judgment of drugs, and specialized technical sub- comm, Bioequivalence certificate review, Pricing: the CIF (Cost, Insurance and Fitters, Stability committee to assess drug stability, Packaging & naming committees) (price in importation contract), Pharmacoeconomic committee judges the cost-effectiveness of the new drug.”* said an owner of a TOLL company

**_According to the technical capacity of committee only 70-80 components/month could be investigated**: *"The only solution that the committee adopted for that delay was to increase factory licensing fees from LE 5000 according to law to LE 50000 & drug registration fee from LE 300 to 15000 (further increase occurred to 50000 since mid-2016)"* said an ex-manager of CAPA.

**Views of policymakers toward different pricing policies:**

*_"The cost of drug content is as small as that of a bonbon". "Even more, the package cost may be more costly than the drug content,"* said a member in an international pharmaceutical research organization.

_By asking about cost of raw material: *“it represents 10-50% of cost of generic and that a new ministerial decree (MD) in 2013: 1st6 registered items in a box have a price of 65% of the reference price then the next 4-5 registered items in a box have 60% of reference price…We have a lot of qualified experts & the cost of manufacturing raw materials is not high but what about people? one of the disinfectants has only one importer, he is about to kill his competitors in the market"* said an owner of a private bioequivalence lab.

**_An interviewee illustrated that there are different types of pricing which are the following**:

*“Value-based pricing: pricing depends on value & also on willingness-to-pay. Value depends on the value of QALY in that country. The budget impact is put into consideration. Internal reference: for generic drugs. It decreases by 10% for every new generic one. Also, the

“External reference pricing”: when a country's price is determined according to the least, average or highest price present in a certain basket of countries. The reference price can be the factory, retail or the public price. Egypt’s reference price is the public one. Because Egypt, for example, is in the same basket of Germany, this stimulates Germany to postpone approving the introduction of an on-patent drug into the Egyptian market because the low prices in Egypt will have its percussion in Germany. Therefore, Egypt has to import this drug despite being that expensive.”* said an ex-manager of CAPA.

**_An interviewee highlighted that the public price of a drug is categorized as follows:** *“30-60% production cost, 40%: 25% as a profit margin for the pharmacists, 10% as a profit margin for the distributor 5% as a profit deductible for medical profession club. This means that for some drugs the marginal profit does not justify drug production.”* said a member of drug industrial chamber

_She added: There are 3 possible solutions:

*“1-National social health insurance.2- Provide Dollars to pharmaceutical investors at a special price of many drugs.3-Selective re-pricing of underpriced drugs that record a financial loss either by raising a small percent for all drugs or by raising a group each year.”*

**_One of the interviewees gave an example**:" *an anti-constipation tablet is sold by public companies at 60 piasters has a substitute sold by private companies at LE 30, the contraceptive pills at 65 piasters versus LE 25.* *"Patients claim that it is better to have more expensive drugs provided being available".* said a member of a public pharmaceutical company.

**_An interviewee said** **about drugs shortage: "***according to the last monthly report on missing drugs, those missing with available substitutes are 932 drugs, which include 370 drugs with a price below 10 pounds. Those missing but without substitutes are 904 which include 348 with a price below 10 pounds. CAPA decided to increase the price as it costs the company. One week after the re-pricing decision the drug was available in the market but media and syndicate of pharmacists had great objections.”* said a member of the NMRA.

_One of the interviewees mentioned that *“shortages are found in 300 drugs out of 13000, mainly in laxatives, albumin & anti-Rh serum, some missing drugs have no substitutes, others have more expensive ones. It has been noticed that missing drugs are either very*

*cheap drugs or imported ones. The latter have a marginal profit of only 6%, therefore they are unable to tolerate a big increase in exchange rates.”* said a member of drug industrial chamber

_Another interviewee said that “*out of missing drugs only 62 threaten patients' health*.” said a consultant of quality control in private pharmaceutical company

_A member of the public pharmaceutical company said that *“according to Egyptian laws an increase/decrease in the exchange rate of Dollars must be accompanied by re-pricing of drugs so that the companies carry only 50% of the effect of this change.”*

_Another interviewee added that: *“pricing of most drugs has occurred when a Dollar was exchanged for 3-4 pounds. The only increase (of 20%) occurred on May 2016 when a Dollar was exchanged for 8.8 pounds. Now an additional big change in currency rate has occurred.*” said an ex-manager of CAPA.

**Views toward pharmaceutical regulation:**

_A member in NMRA stated that: *"inspection is done for factories in the form of Random selection of a pilot batch to be analyzed in central governmental labs.-Another inspection of equipment & GMP certificate is scanned-and finally microbiology inspection".*

_By asking another interviewee **about counterfeit drugs**, he said that *"Adulterated drugs is a slang expression. Usually, they are imported drugs unregistered for fear of unsatisfactory pricing. They fill in the market space for a missing drug. Less frequent come drugs with weaker API, Egypt is the gate for many unregistered drugs to Africa"* said by a member of a public pharmaceutical company*. _"Many of unregistered drugs are sexual drugs and tramadol group. Solving the problem is by coding the package by a barcode system & supporting inspectors by security team & transportation facilities.*" said a consultant of quality control in private pharmaceutical company

_Another interviewee highlighted that *"counterfeit drugs represent 20% of market drugs and drug trafficking is done also for anticancer & cardio surgery drugs for fear of registration & low pricing. Counterfeit drugs worldwide cost $ 200 Billion, in Egypt, there are 1600 drugs."* said a WHO consultant.

_Another interviewee explained that*:" other than unregistered drugs are those manufactured out of formal factories. These are present mainly in rural & remote areas and in vitamins, otherwise, the market expels them quickly "e.g. a non-effective anti-platelet that costs 400 pounds is refused by doctors"*. said a member of drug industrial chamber

*_"Chain pharmacies, private single pharmacies & even individuals sell unregistered drugs. I know a pilot who buys drugs from western counties & sells them in Egypt for a profit of 30%"* said an owner of a private bioequivalence lab.

**_As concerning Pharmaco-vigilance**, an interviewee mentioned that:" *there are training fees for private companies personnel about 10000 pounds held by MOH personnel & income (50%) is for the assistant minister"* said a member in the registration committee.

_Another interviewee said that:*" there was unit establishment in CAPA in 2010 for training of pharmacovigilance coordinating personnel of companies on occurrence of any side effect of already marketed drug, they should immediately report to PVU in CAPA which immediately reports to WHO office in Upsala in Sweden as done by all its country offices"* said an ex-manager of CAPA.

**_On asking an interviewee about the effectiveness of MOH & HIO drugs in comparison to private market drugs,** she answered, *"I don't know, on chemical analysis of pilot batches all are the same, but this difference could have resulted from the effect of adding excipients or bioequivalence or the low grade of imported raw materials".* said a consultant of quality control in private pharmaceutical company

***_As regards transparency inside NMRA*** *she added "(a) why does NMRA accept to reanalyze a failing drug by analyzing a new pilot batch, when the latter succeeds which order will be released in the market? (b) when a batch does not pass according to NMRA labs it is allowed to repeat the analysis in an external lab, and it may pass. (c) The inspection department personnel of CAPA are responsible for the random selection of the batch. Randomization is not guaranteed. (d) The head of NMRA took a decision that exempts chemotherapeutic drugs from NMRA analysis arguing for the lack of professional safety requirements in NMRA labs. Some told that this decision has been taken for the sake of private companies. (e) Establishment of a committee inside NMRA "The Non-conformity committee" that requires a majority voting to decide for rejection of an already nonconforming drug by the lab.*"

**Views toward the research and development role**

*_"Among the multiple roles of NMRA it has a department for innovative trials of raw material synthesis.* *In Egypt there are many patents regarding raw material synthesis, nevertheless most raw materials are imported”*. said a research consultant in a private pharmaceutical company.

_One of the interviewees highlighted that: "*the National Research Institute has better resources to conduct research. There, teams are bigger & multidisciplinary. We publish international research that comprises our innovative ideas; these ideas can be picked up by any foreign reader. Locally, these ideas need 10-15 years till the marketing phase"* said an academic professor in the faculty of pharmacy.

_He also added that:" *there is no great need for planting herbs to replace imported raw materials. Biosynthesis is an easier method. We could synthesize some simple drugs in the labs of the school of pharmacy during study years".*

_ A member in drug industrial chamber said: *"I consider not having a law for clinical trials as a scientific crisis. This hinders both industries & exporting. The importing country requires from Egypt a GMP certificate, the market price in Egypt is the country of origin and a bioequivalence certificate which is an evidence of the effectiveness of the new generic drug after being administered. Labs for bioequivalence in Egypt are not accredited, hence the exporter makes the study in accredited labs outside Egypt e.g. is Jordan which costs him more expenses & is paid in Dollars."*

_One of the interviewees said that:" *we need a research center that coordinates between research centers in Egypt. Duplications in research activities occur and dissociation form market needs are found [My American professor told me on visiting Egypt "You are not poor but you have a problem with coordination]*" said a consultant of quality control of pharmaceutical companies.

_He further added that:" *worldwide only 2-3 originator drugs appear yearly so we depend on generic drugs. For generic drugs to be like innovator drug the Research &Development departments should work hard and this is finally confirmed by bioequivalence study*."

**Views toward drug promotion**

_One of the interviewees mentioned that:" *there is a great slope & deterioration in the aspect of the relationship between pharmaceutical companies & physicians. (Dirty business) appear in the form of: [A monthly meal-An annual conference abroad-Renovation of his private clinic-Mobile phone]"* said an owner of a TOLL company.

One of the suggestions for control as mentioned by the interviewee is to use generic drugs (non-proprietary names) (NPN).

**Views toward medicines production capability in the country:**

_A member in drug industrial chamber said *"Drug industry is very old in Egypt, Egyptian pharmacopeia is one of oldest; nonetheless we currently use US & British pharmacopeia. we have 600 factories including cosmetic companies/1200-1500 TOLL, nevertheless, we import even from Gulf countries."*

_One of the interviewees explained that:" *the pharmaceutical industry started in Egypt in 1937, the first factory was named Egypt factory. The first Arabian law for pharmaceuticals was 127 for 1952 which was a model for other Arabian countries to establish their drug regulatory systems."* said a parliament member

**TOLL Companies:**

They are establishments of pharmaceutical companies that started in 1990. A TOLL company is a company that contracts one or more pharmaceutical companies to manufacture its registered drugs. Both companies are labeled on drug package, they are about 600 companies.

_An owner of a TOLL company mentioned certain production hinders*: "At registration: [1] a limited number of boxes (two) are allowed out of the 12 boxes for each molecule.[2] A need for more transparency: Communication using a single mail. Each claim fee costs 1000 LE for initial acceptance for registration of a certain molecule. [3] Delayed registration process that may last for 3 years "I need to work at the same time on 10 medicines to buffer for losses &delays in some of them".*

**Industry (General)**

_An interviewee said that:*" >95% of raw materials are imported "why we don’t use biotechnology in producing synthetic raw materials"* said a member of the registration committee*.*

_One interviewee said *"Raw materials have many grades, hence the quality of products is variant. Chinese raw materials have two grades (high → USA) and (low → Egypt)"* said an owner of a private bioequivalence lab.

**As regards Importing**, an interviewee explained that*:" before the decrease in the credit rating of Egypt on the international level, the pharmaceutical companies exporting drugs to Egyptian importing companies were accepting deferred payment based on the availability of a bank account in dollars, but now cash payment is conditioned.*" said a member of NMRA.

_Banks' timing in opening such accounts is too much delayed *"How do Egyptian banks not consider pharmaceutical needs as a priority"* said a member in a public pharmaceutical company.

*_"Also importers are not allowed to pay the due dollars from the parallel market, but only form banks. The latter again do not provide enough dollars so that the imported batches suffer delayed custom release with increased custom fees and tariffs."* said an owner of a TOLL company.

_A member in drug industrial chamber suggested solving the problem of delays in bank supply of dollars by:" *estimating the dollar needs of pharmaceutical investors at the beginning of the fiscal year from data provided by Drug Industry Chamber. This bulk should be presented for drug industry & importing from the beginning of the year".*

**
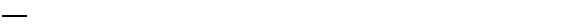
II- Perspectives of physicians towards determinants of patients' access to essential drug list:**

**a- Prescribing behavior of physicians**

_Most of physicians mentioned that: "*effectiveness, contraindication, cost of medicine constituted the basic criteria for selecting a drug during prescription"*.

_Most physicians illustrated that the most common sources that they take into account in justifying their prescription choices were from the internet followed by asking their senior colleagues and sometimes reading from a textbook.

_ All physicians interviewed said that*:" I never respond when pressured by patients to prescribe non-indicated drugs*" except only one female physician said that*: "I respond to that pressure to avoid the headache of elongated discussions"*

**b- Perception about Affordability**

_One female physician said that:" *I'm instructed by MOHP to prescribe only 40 packages of EDL per month*"

*_*Giving the patients free samples, choosing the cheaper alternative, taking more than one ticket and sometimes personal aid was highlighted by almost all interviewed physicians as actions that they take when the patient complains about the cost of medication.

_A male physician mentioned also that*: "Referring the patient to social affairs unit to get benefit from the different social support methods (e.g takafol&karama pension) or non-governmental organization (e.g Resala) after writing a report about his/her case".*

**c- Attitudes of Physicians towards Generic Drugs Prescribing**

_There was a consensus among physicians that the quality of drugs depends on the manufacturing company.

_All physicians said that: "*it is feasible to implement a prescribing system based on the international non-proprietary name (INN) but needs monitoring and training", the benefits of such a system that it makes medicine in public sector different from that of a private one."*

**d- Relation to Medical Representatives**

_All interviewed physicians mentioned that*:" we are rarely affected by motivation by medical representative sometimes we only benefit from their information on the medicine if it is new but when there is nothing new we don't pay attention e.g mouth wash all are the same."*

**e- Perception of EDL Policies**

_All physicians reported that among the benefits of restriction of drug prescription to EDL is the reduction of financial load on patients, one male physician also said: "*this makes medicine is available to a larger number of patients and ensures the commitment of patient to the treatment and decrease confusion between the multiple names of drugs.*"

**f- Perception and Commitment to Guidelines Policies**

_By asking physicians about their knowledge about standard treatment guidelines, only two mentioned that it is not present and the other two said there are Integrated Management of Childhood Illness (IMCI) guidelines only, they are available on daily basis and they always make use of it and they receive training on these guidelines which is very effective.

**g- Managerial control on prescribing behavior**

_Two physicians mentioned that:" *There is monitoring on prescribing behavior e.g writing by generic name but this monitoring is not effective [just paper work]*"while other physicians mentioned that there is no monitoring on their prescribing behavior but they internally arrange together to limit the number of prescriptions to one or maximum two medicines.

One male physician said, "*It is allowed to give the emergency treatment with no limited number*".

**h- Perception about positive aspects of Pharmaceutical Management in PHC:** _Almost all physicians said that: "*easy intake =dispensing of medicine is among the positive aspects of pharmaceutical management in the PHC system.*"

**i- Perception about negative aspects of Pharmaceutical Management in PHC:**

_Most physicians mentioned that:" *quantity of medicine is not enough and not all dosage forms are present.*" One male physician highlighted "*if antibiotics are more available it will be better*". Another female physician said, *"we need better analgesics and antibiotics and antifungal treatment is not available and injections are not enough".*

_One male physician said*: "Those who are not included in the family medicine folder pay all the cost of the medicine otherwise they pay part of the cost".*

_Another female physician mentioned that: *"Sometimes PHC employees, workers, nurses and even doctors take drugs without permission."*

**III- Perspectives of pharmacists towards determinants of patients' access to essential drug list:**

**a- Knowledge about the EDL:**

_All pharmacists know that this is the list of drugs that should be available in the facility according to the common health problems in the community and this list is procured by MOH through bids then they choose the least price offered by companies.

**b- Existence of functioning mechanisms to improve the dispensing practices**

_All pharmacists mentioned that they follow the proper dispensing practices but there is no monitoring. Two of them said *" Monitoring is conducted only on dispensing practices when there is a problem e.g rate of dispensing increased for a certain drug like in winter antibiotics dispensed for patients increase"* and by asking about if there is training on these practices, they said: "*There is no training"* They know these practices by their experience when they work in private pharmacies.

**c- Existence of drug committee.**

_All Pharmacists in one PHC unit said that*:" There is meeting every month in this committee but aims to adjust [ legal paper work] ",* Basically, aim of this committee is to know what should be written on the prescription e.g treatment is suitable for the diagnosis "*Many times diagnosis written by physician is not matching with medicine prescribed like antispasmodic prescribed for vomiting and if the pharmacist commented on that diagnosis he get in clashes with the physician*. "

_Another pharmacist said*:" When there was an inspection by someone in charge, fabrication didn't happen"*

_While in the other PHC unit, drug committee holds meeting with pharmacists and physicians but not on regular basis to demonstrate what are drug interactions, for explaining that there was shortage in a certain drug and now it is available or a new drug is added to the list and they speak with physicians about indication, side effects and so on.

**d- Quality of generic versus brand:**

_All Pharmacists in both PHC units said that*:" There is no difference but it depends on the manufacturing company. Certain pharmaceutical companies have quality products"*

**e- Percent of prescribed medicines from the EDL:**

_Almost all pharmacists in the rural unit mentioned that it is 90-100%, one of the pharmacists demonstrated that:" *There are instructions not to prescribe from outside the list and that one colleague was previously punished because he prescribed medication outside the list".*

_But in the urban unit, all pharmacists said it is 50%-60% "*Many are prescribed from outside the list as there is a shortage in many drugs"*

**f- Percent of medicines prescribed by generic name:**

_One pharmacist mentioned that:"*Percent of medicines prescribed by generic name is 80-100% (physicians are given instructions to prescribe by generic name)",* while another pharmacist in the same facility said "*very small percent of physicians prescribe by generic as it is easier for physicians to prescribe by brand name e.g it is easier to recall Flagyl, not metronidazole" and this makes the pharmacist sometimes get in trouble with the patient as patient cannot understand that so the patient insist to have the drug name he is familiar with*"

_One pharmacist justified that "*It is easier for physicians to write by brand name and difficult to recall generic name e.g it is easier to recall augmentin from amoxicillin plus clavulanic acid"*

**g- The percent availability and average period of stock-outs of unexpired essential**

**tracer medicines**

_All pharmacists in both rural and urban units agreed that some medicines should be present all the time in the unit e.g antihypertensive, antidiabetic, antipyretics, antibiotics, cough sedatives, milk formulas, and drugs of antenatal care like folic acid, iron.

_All pharmacists mentioned that they are asked to calculate their needs by taking the average of three consecutive months and there is a computer program to calculate quantities needed for each facility. In the rural facility, they order every month. While in the urban unit they order every three months as transportation fees are paid by pharmacists themselves"100-150 LE".

_One pharmacist in the rural facility illustrated that "*Stock increased by 10% e.g if 100 bottles are required per month I order 110 to avoid stock out"*, another pharmacist in the same facility mentioned that*: "There was no stock out in the previous month*".

_One pharmacist in the urban unit said*: "There is a shortage of antibiotics in general as most of the physicians prescribe antibiotics."*

_Another pharmacist in the same facility mentioned that: "*There was stock out of penicillin(3 packages only) and anti-diabetic medicine in the previous 3 months".*
