# Supplementary material for: Perspectives of pharmaceutical stakeholders on determinants of medicines accessibility at the primary care level
Source: J Egypt Public Health Assoc. 2021 Jan 13;96:1. doi: 10.1186/s42506-020-00062-x (PMC7806678; doi:10.1186/s42506-020-00062-x)
Supplement: Supplementary file 2 — Additional file 2. Suggestions of key informants for improving access to medicines. Suggestions of physicians and pharmacists for improving drug affordability. [file 42506_2020_62_MOESM2_ESM.docx]

**Appendix 2:**

**Suggestions of key informants for improving access to medicines**

1. Enforce continuous medical education in NODCAR, being weak due to lack of financial resources “Experience is hard to get as knowledge & experience are concentrated mainly in formal projects funded by national or international budget”.
2. It is easy to manufacture generic drugs. “There is no need to manufacture innovator drugs being hard, costly & needs 10-15 years till its market release".
3. Strengthen coordination between different research organizations such as universities, National Research Institute, NODCAR & Atomic Energy Agency.
4. Complete separation between regulatory agencies and private sector to avoid conflict of interest.
5. Ensure professional safety requirements, being tackled in NODCAR despite frequent complaining of personnel.
6. Infection control & its allowance
7. Health insurance coverage is also demanded.
8. Design & support a research plan
9. Support decisions that promote health of Egyptian population.
10. Establish factories for raw materials’ manufacturing & also exepients but without monopoly. To produce (API) active pharmaceutical ingredient we need to conduct a joint venture with India or China to be led by well-selected well-paid experts.
11. To be practical & feasible we better start with a small number of factories e.g. for antibiotics with highest utilization rate in Egypt.
12. Control for situations of conflict of interest, strengthen transparency.
13. Proper pricing meanwhile subsidize poor patients: “Freely dispensed MOH drugs can be manufactured by few selected factories, while other factories are left to be affected by market forces to provide affordable drugs for those who are able to pay.
14. Transparency in control agencies of CAPA.
15. Support small entrepreneurship & young investors through facilitation in allocating land and facilitation by elongation of the period allowed before establishing a factory
16. Strengthen control measures on drugs before, during & after manufacturing.
17. Emphasize legibility of marketing strategies used.
18. Majority recommend to use generic drugs.

19- Tax reduction on few selected or even all pharmaceutical factories.

20- Establish EDA as in USA, Somalia, in Saudi Arabia & in Jordan. By its establishment Egypt can duplicate the amount of imported drugs. Jordan has increased it by four times after its FDA establishment.

21- Financial support of companies with long-standing debts.

22- For success of established EDA we need to delegate different tasks to qualified highly skilled & highly specialized individuals "My MD thesis in USA was about serotonin, each supervisor of the thesis was concerned only with a small part in which he is highly specialized". On the contrary, in Egypt we don't delegate with matching qualifications & experience. Even Europe is not as strict as the USA in this regard.

23- The structure of proposed EDA should include CAPA, NODCAR & NORCB. NODCAR is for research & lab analyses of drugs, cosmetics & devices while NORCB does the same roles for biological & vaccines.

24- Factors behind success of Jordan in drug industry & importation are proper pricing, accreditation of bioequivalence labs (from EU) & finally small number of well-educated well-coordinated pharmacists.

**Suggestions of physicians and pharmacists for improving drug affordability**

1. Increase quantities of medicine.
2. Try to return to the old system that patient take the medicine free.
3. There should be an enforcement to prevent patients who don't need the medicine from taking it-if there is a nurse that help the doctor to explain what are the drawbacks of taking a medicine that is not needed as the doctor at most of the time is overloaded.
4. Increase local production of quality drugs e.g in the past there was an imported anesthetic but was expensive and with time an Egyptian company "art pharma-dent" produced it by about half the price.
5. increase fees of the ticket from 1 pound to 5 pounds
6. increase medications for vaginal bleeding, creams and suppositories for vaginal infection as there are many cases and its treatment is expensive "there is shortage in ceprofar"
